# Comparative Performance of ^68^Ga-PSMA-11 PET/CT and Conventional Imaging in the Primary Staging of High-Risk Prostate Cancer Patients Who Are Candidates for Radical Prostatectomy

**DOI:** 10.3390/diagnostics14171964

**Published:** 2024-09-05

**Authors:** Guido Rovera, Serena Grimaldi, Marco Oderda, Giancarlo Marra, Giorgio Calleris, Giuseppe Carlo Iorio, Marta Falco, Cristiano Grossi, Roberto Passera, Giuseppe Campidonico, Maria Luce Mangia, Désirée Deandreis, Riccardo Faletti, Umberto Ricardi, Paolo Gontero, Silvia Morbelli

**Affiliations:** 1 Nuclear Medicine Division, Department of Medical Sciences, University of Turin, 10126 Turin, Italy; 2 Nuclear Medicine Division, AOU Città della Salute e della Scienza di Torino, University of Turin, 10126 Turin, Italy; 3 Urology Unit, Department of Surgical Sciences, AOU Città della Salute e della Scienza di Torino, Molinette Hospital, University of Turin, 10126 Turin, Italy; 4 Department of Oncology, Radiation Oncology, University of Turin, 10126 Turin, Italy; 5 Nuclear Medicine Division, Gustave Roussy, 94805 Villejuif, France; 6 Radiology Unit, Department of Surgical Sciences, University of Turin, 10126 Turin, Italy

**Keywords:** prostate cancer, hormone-sensitive prostate cancer, positron emission tomography, PSMA PET, primary staging, conventional imaging

## Abstract

This prospective study aimed to (1) compare the diagnostic performance of ^68^Ga-PSMA-11 PET/CT with respect to conventional imaging (computed tomography (CT) and bone scintigraphy (BS)) in the primary staging of high-risk prostate cancer (PCa) patients and (2) validate PSMA-PET/CT accuracy in pelvic nodal staging in comparison with postoperative histopathology and assess PSMA-PET/CT’s impact on patient management. Sixty castration-sensitive high-risk (ISUP 4–5 and/or PSA > 20 ng/mL and/or cT3) PCa patients eligible for radical prostatectomy were enrolled (median PSA 10.10 [IQR: 6.22–17.95] ng/mL). PSMA-PET/CT, compared with CT, identified nodal (N) and/or distant metastases (M1) in 56.7% (34/60) vs. 13.3% (8/60) (*p* < 0.001) of patients: N + 45% vs. 13.3% (*p* < 0.001), M1a 11.7% vs. 1.7% (*p* = 0.03), M1b 23.3% vs. 1.7% (*p* < 0.001). Compared with BS, PSMA-PET/CT localized unknown skeletal metastases in 15% (9/60) of cases, with no false negative findings. Overall, PSMA-PET/CT led to a TNM upstaging in 45.0% (27/60) of cases, with no evidence of downstaging, resulting in a change in management in up to 28.8% (17/59) of patients. Compared with histopathology data (*n* = 32 patients), the per-patient accuracy of PSMA-PET/TC for detecting pelvic nodal metastases was 90.6%. Overall, the above evidence supports the use of PSMA-PET/CT in the diagnostic workup of high-risk prostate cancer staging.

## 1. Introduction

The accurate staging of prostate cancer (PCa) is essential for treatment planning in high-risk patients. Radical prostatectomy with or without lymphadenectomy and definitive radiotherapy (RT) are the main curative treatments for PCa. However, a significant proportion of patients may experience disease recurrence following these interventions. A more accurate disease staging could prove beneficial in enhancing initial treatment, subsequently reducing the risk of relapse. Until recently, clinical guidelines have recommended the use of conventional imaging (CI) techniques such as computed tomography (CT) and bone scintigraphy (BS) for the primary staging of high-risk PCa; however, such techniques have suboptimal accuracy. In this context, the novel radiopharmaceuticals for molecular imaging which target the prostate-specific membrane antigen (PSMA) have gained traction. PSMA is a transmembrane glycoprotein constitutively expressed within the apical epithelium of prostatic secretory ducts and overexpressed in PCa cells, in which it migrates to the luminal surface as malignant transformation occurs.

In the past years, PSMA positron emission tomography/computed tomography (PSMA-PET/CT) has become an established imaging modality for restaging patients with early biochemical recurrence or persistence [1,2,3,4,5]; indeed, the European Association of Urology (EAU) guidelines recommend performing a PSMA-PET scan in patients with PSA failure after radical treatment, provided that the imaging data can potentially impact the patient’s clinical management [6]. More recently, literature studies have demonstrated the higher sensitivity of molecular imaging with PSMA PET/CT compared to conventional imaging in the setting of the primary staging of high-risk PCa [7,8]. Accordingly, the revised EAU guidelines recommend performing PSMA-PET/CT imaging (if available) in the primary staging of intermediate and high-risk PCa patients [6]. However, in the absence of clear data regarding the ideal management and prognosis of patients staged with PSMA-PET/CT, they advise caution when basing therapeutic decisions on molecular imaging findings, especially in cases of patients with metastases detectable only by PET/CT. Therefore, more studies evaluating the diagnostic performance of PSMA-PET/CT and its impact on patient management are needed. This prospective study, which reflects real-world practice, aimed to evaluate the ^68^Ga-PSMA-11 PET/TC diagnostic performance in the primary staging of patients with high-risk PCa compared to conventional imaging modalities, as well as to assess its potential impact on patient management. Furthermore, the diagnostic accuracy of ^68^Ga-PSMA-11 PET/CT in pelvic nodal staging was assessed using postoperative histopathology data as a reference standard.

## 2. Materials and Methods

### 2.1. Study Design

As part of a prospective study, sixty (*n* = 60) consecutive PCa patients underwent whole-body ^68^Ga-PSMA-11 PET/CT imaging at our institution (Department of Nuclear Medicine, University Hospital of Turin) between April 2021 and January 2024. The patients were enrolled according to the following inclusion criteria: (1) histologically proven diagnosis of PCa; (2) high-risk classification according to the d’Amico criteria (ISUP 4–5 and/or PSA > 20 ng/mL and/or cT3); (3) eligibility for radical prostatectomy (i.e., ≥10 years life expectancy and no major contraindication for radical prostatectomy); and (4) CT and BS performed within three months of ^68^Ga-PSMA-11 PET/TC. The exclusion criteria were (1) an inability to undergo a PET/CT scan; and (2) previous treatments, including androgen deprivation therapy (ADT).

### 2.2. Procedures and Image Interpretation

The radiopharmaceutical (^68^Ga-PSMA-11) was synthesized in the radiochemistry laboratory of the Division of Nuclear Medicine of the AOU Città della Salute e della Scienza, University of Turin, as previously documented [9], in accordance with procedural guidelines [10,11]. ^68^Ga-PSMA-11 was administered intravenously (1.8–2.2 MBq/kg) to all patients, followed by hydration with 0.5 L of saline solution during uptake. Informed consent was obtained from all subjects before administration. The diagnostic imaging did not require specific patient preparation. ^68^Ga-PSMA-11 PET/CT imaging was performed according to standard recommendations, as previously described [9], using a digital PET/CT scanner (Vereos, Philips HealthCare, Eindhoven, The Netherlands). PET emission data were co-registered with a low-dose CT scan for attenuation correction and reconstructed with the ordered subset expectation maximization (OSEM) algorithm (3 iterations, 15 subsets). Two experienced nuclear medicine physicians independently reviewed the PET/CT images, and any resulting discrepancy was solved by consensus. In accordance with the E-PSMA procedure guidelines [10,11], a per-region analysis was performed. Pathological findings were defined as areas of increased focal radiopharmaceutical uptake compared to the background, not localized in sites of known physiologic uptake.

A contrast-enhanced diagnostic CT scan of the abdomen and pelvis was acquired using a 64-slice CT scanner (Optima, GE Healthcare, Milwaukee, WI, USA). In accordance with the current protocol recommendations of the Italian Society of Medical and Interventional Radiology (SIRM), CT scans were acquired with a voltage level up to 120 kV based on the patient’s BMI and a dose-modulated tube current (automated mAs). A contrast volume (Iomeprolo 400 mg/mL) up to 0.63 gI/Kg was injected at 2.0 mL/sec, and imaging was performed approximately 70–90 s after contrast injection for the portal venous phase. CT images were reconstructed with the slice thickness down to 1 mm. The images were analyzed using a dedicated workstation (Advantage Windows, General Electric, Boston, MA, USA).

Bone scintigraphy was performed using a gamma camera (Discovery NM/CT 670 ES, GE Healthcare, Milwaukee, WI, USA), equipped with low-energy high-resolution (LEHR) collimators and an energy window centered at 140 keV ± 10%. According to the procedural recommendations of the Italian Association of Nuclear Medicine (AIMN), 2–4 h after the intravenous administration of 300–740 MBq of ^99m^Tc-HMDP, whole-body planar images in anterior and posterior projections were acquired with a scan speed of 12–15 cm/min and a matrix size of 256 × 1024 pixels. Additional static or tomographic imaging (single-photon emission computed tomography SPECT) of areas of interest was performed according to clinical evaluation, and SPECT data were co-registered with a low-dose CT scan for the attenuation correction and anatomical localization of scintigraphic findings. The images were analyzed using the Xeleris v4 software (GE Healthcare, USA).

In this prospective observational study, clinical decisions were based on conventional imaging (CT and bone scintigraphy) according to guidelines. However, a retrospective evaluation of the PET-driven change in management was performed by assessing the potential impact of PET imaging on the subsequent patient management, taking into account the additional findings from molecular imaging. The change in management criteria were defined as follows: (a) switch to systemic therapy due to previously unknown metastatic involvement; (b) change in the lymphadenectomy template in patients who are candidates for surgical treatment; (c) modification of the RT treatment planning and/or hormonal treatment; (d) potential stereotactic ablative RT (SABR) treatment in the case of the novel identification of oligometastatic disease spread; and (e) identification of collateral PSMA-avid oncologic findings (unrelated to PCa). Finally, the presence of false positive findings solely at conventional imaging (i.e., bone scintigraphy) was recorded, since in this case, staging with PSMA-PET/CT could obviate the need for further confirmatory tests, thus impacting patients’ management.

### 2.3. Statistical Analysis

For each patient, the collected data included information about age, multiparametric magnetic resonance imaging (mpMRI) findings, disease staging at conventional imaging (CT and BS), prostate biopsy and histopathological grading, PSA measurements, findings at PSMA-PET/CT imaging, and postoperative histopathology data. Anonymized data regarding the patients’ clinical features and imaging findings (PSMA-PET, CT, BS) were stored and queried using a relational database [12].

Population characteristics were presented as absolute/relative frequencies for categorical variables and as a median (Inter Quartile Range (IQR)) for continuous ones. Inferential statistics were performed using the Mann–Whitney test for continuous covariates and Fisher’s exact test for categorical ones, respectively. CT and PSMA PET/CT detection rates were compared using McNemar’s exact test. PSMA PET/CT’s accuracy in detecting pelvic nodal metastases was compared with histopathology in terms of sensitivity, specificity, positive predictive value (PPV), negative predictive value (NPV), and accuracy.

All reported *p*-values were two-sided, at the conventional 5% significance level. The data were analyzed as of April 2024 using IBM SPSS Statistics for Windows, version 26.0 (IBM Corp., Armonk, NY, USA).

## 3. Results

### 3.1. Study Population

Sixty (*n* = 60) high-risk PCa patients were enrolled in this prospective study and underwent preoperative staging for nodal (N) and distant metastases (M1) with computed tomography, bone scintigraphy, and ^68^Ga-PSMA-11 PET/CT. The median age of the cohort was 73 years (IQR: 68–76 years). The median PSA at diagnosis was 10.10 ng/mL (IQR: 6.22–17.95 ng/mL), with comparable values at the time of PET/CT imaging (10.51 ng/mL, IQR: 6.50–21.00 ng/mL). At preoperative multiparametric magnetic resonance imaging (mpMRI), a PI-RADS 5 score was reported in 83% (39/47) of patients, the median maximum lesion diameter was 20 mm (IQR: 15–29), and a suspicion of extra-prostatic tumor extension (≥cT3a) was reported in 46.8% (22/47) of cases. Histopathological grading at biopsy revealed an ISUP grade ≥4 in 80% (48/60) of patients. The population characteristics are summarized in Table 1.

### 3.2. PSMA-PET/TC Findings

According to the molecular imaging TNM (miTNM) classification [13], pathologic pelvic lymph node metastases (miN) were detected in 45% (27/60), extraregional lymph node metastases (miM1a) were detected in 11.7% (7/60), and skeletal metastases (miM1b) were detected in 23.3% (14/60) of the cases. No visceral metastases (miM1c) were detected. Overall, 43.3% (26/60) of patients were found to be non-metastatic (N0M0), while 25% (15/60) had solely pelvic lymph node metastases (N), and 31.7% (19/60) had distant metastases (including 5/19 M1a, 12/19 M1b, and 2/19 M1a+b). Oligometastatic disease spread (≤3 lesions) was detected in 40% (24/60) of patients, while multimetastatic involvement was detected in 16.7% (10/60). Among the 143 pathological findings identified at ^68^Ga-PSMA-11 PET/CT imaging, PSMA-RADS scores [10] were distributed as follows: 30.1% (43/143) were classified as PSMA-RADS 5, 51.8% (74/143) were classified as PSMA-RADS 4, and 18.2% (26/143) were classified as PSMA-RADS 3. All lymph node metastases with a PSMA-RADS score of 3 had a sub-centimeter maximum diameter (median 7 mm [IQR: 4.5–8 mm]).

### 3.3. Comparative Performance of PSMA-PET/CT and CT

PET/CT was positive for regional (N) and/or distant metastases (M1) in 56.7% (34/60) of patients, while CT was positive only in 13.3% (8/60) (*p* < 0.001). The PET/CT detection rate was higher than the CT detection rate in all anatomical regions: pelvic lymph nodes (N1) 45% vs. 13.3% (*p* < 0.001), extraregional lymph nodes (M1a) 11.7% vs. 1.7% (*p* = 0.03), and bone metastases (M1b) 23.3% vs. 1.7% (*p* < 0.001). No patients were understaged by ^68^Ga-PSMA-11 PET/CT compared to CT; on the contrary, molecular imaging led to a TNM upstaging in 50% (30/60) of patients. Specifically, among patients who were non-metastatic according to CT (N0M0, *n* = 52), PSMA-PET/CT identified regional lymph node metastases (N1M0) in 25% of cases (13/52) and distant metastases (M1a/b) in an additional 25% of cases (13/52). On the other hand, among patients with the exclusive involvement of pelvic lymph nodes on CT (N1M0, *n* = 6), PSMA-PET/CT identified new distant metastases (M1) in 66.7% (4/6) of cases. Among patients with newly detected distant metastases (M1, *n* = 17), exclusive skeletal involvement (M1b) was found in 64.7% (11/17) of cases, followed by exclusive extraregional lymph node involvement (M1a) in 23.5% of cases (4/17) and the involvement of both sites (M1a+b) in 11.8% (2/17) of cases. Regarding disease burden, PSMA-PET/CT detected novel oligometastatic and multimetastatic dissemination in 38.5% (20/52) and 11.5% (6/52) of CT N0M0 patients, respectively; furthermore, novel multimetastatic spread was identified in 42.9% (3/7) of patients with CT oligometastatic disease (N1/M1).

### 3.4. Comparative Performance of Bone Scintigraphy and PSMA-PET/CT

Bone scintigraphy detected suspicious bone metastases in 16.7% (10/60) of patients, with oligometastatic involvement in 90% of cases. Suspicious findings at bone scintigraphy were later confirmed only in 4/10 patients. No conclusive pathologic findings were detected solely at bone scintigraphy. On the other hand, PSMA-PET/CT identified novel skeletal metastases (M1b) undetected at BS in 15% (9/60) of patients, with PSMA-RADS ≥4 findings in 66.7% (6/9) of them.

**Figure 1:**
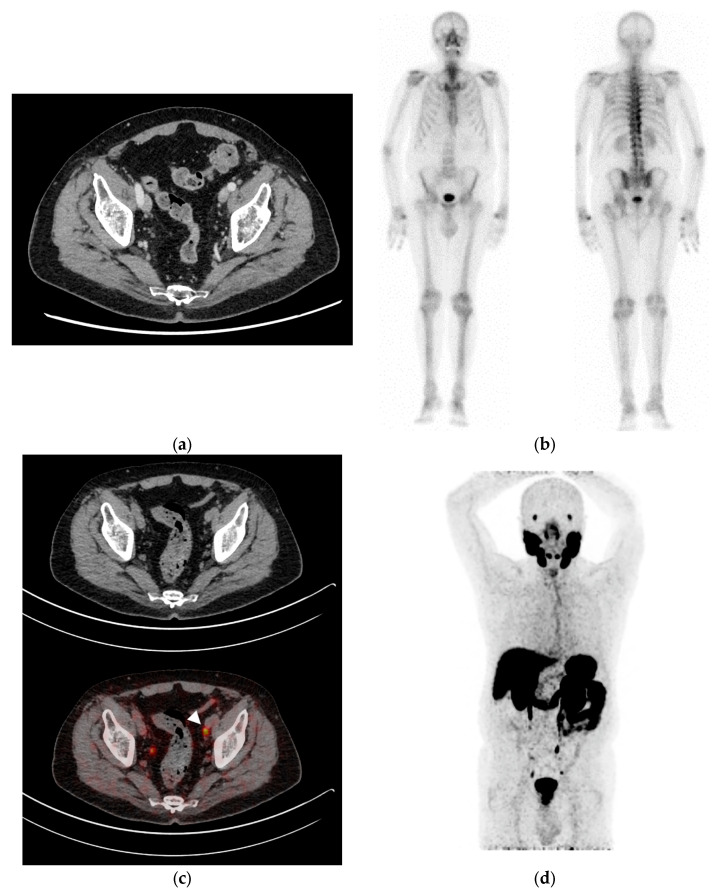
Clinical case: 82-year-old patient, iPSA 11.4 ng/mL, Gleason score 9 (5 + 4), with no pathologic findings at CT (**a**) and bone scintigraphy (**b**). (**c**,**d**) ^68^Ga-PSMA-11 PET/CT: left external iliac lymph node metastasis (5 mm) (arrowhead).

**Figure 2:**
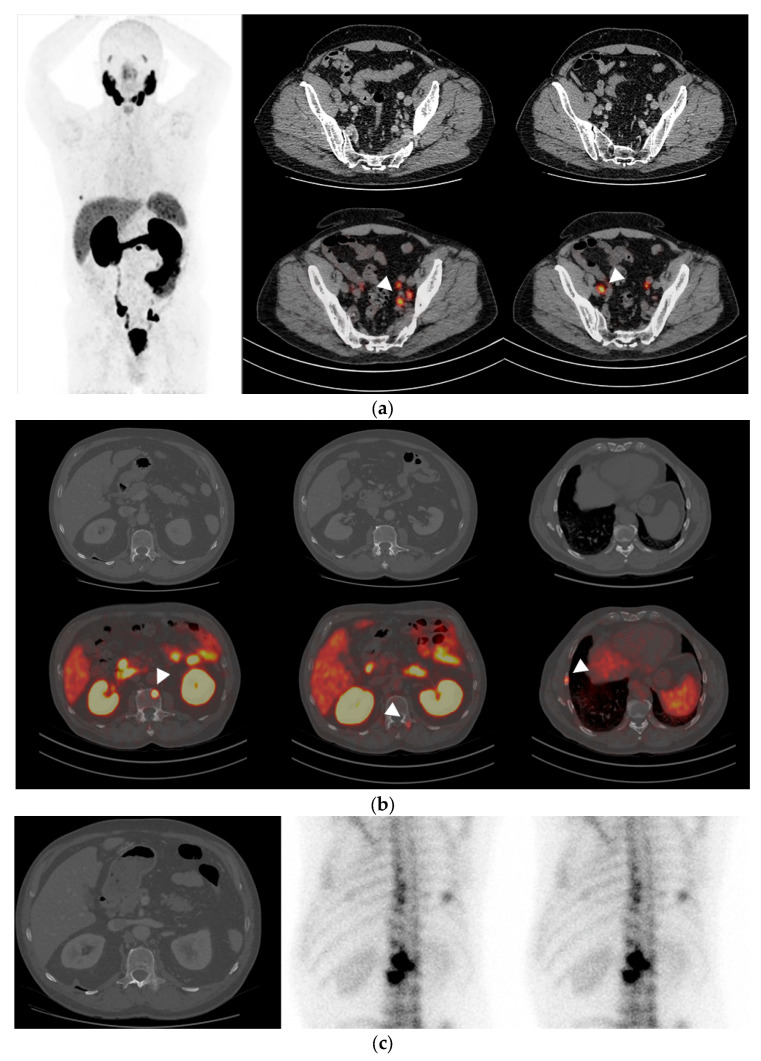
Clinical case: 65-year-old patient, iPSA 28 ng/mL, Gleason score 8 (4 + 4). Multimetastatic nodal (**a**) and skeletal (**b**) dissemination (arrowheads) at PSMA-PET/CT, with negative CT (**a**,**b**) and bone scintigraphy. (**c**) PSMA-PET/CT findings were confirmed at subsequent CT and bone scintigraphy exams performed during follow-up.

Figure 1 and Figure 2 present two clinical cases of metastatic PCa at PSMA-PET/CT undetected at conventional imaging (i.e., CT and bone scintigraphy).

### 3.5. Comparative Performance of PSMA-PET/CT and Conventional Imaging

**Figure 3:**
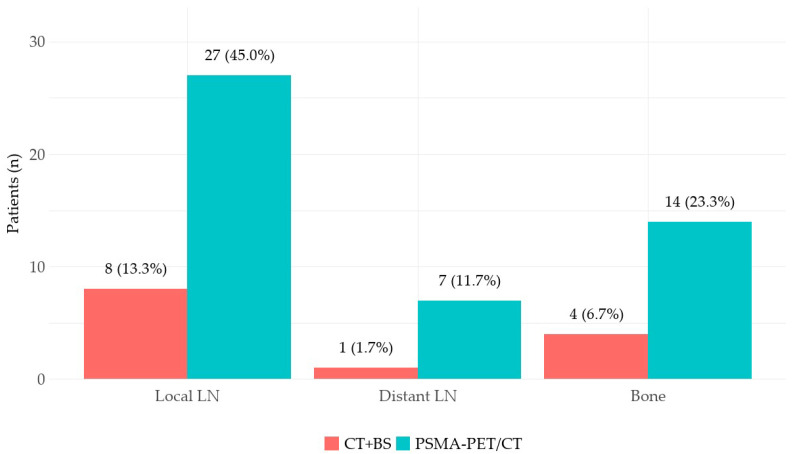
Comparison of the diagnostic performance of conventional (CT + BS) and molecular imaging (PSMA-PET/CT): patients with pathologic findings detected by PSMA-PET/CT and conventional imaging, stratified by anatomical region.

**Figure 4:**
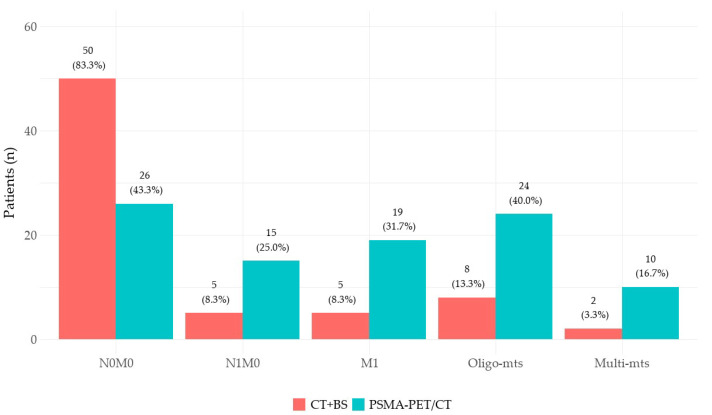
Comparison of the diagnostic performance of conventional (CT + BS) and molecular imaging (PSMA-PET/CT): TNM staging and tumor burden evaluation.

PSMA-PET/CT identified regional (N) and/or distant metastatic localizations (M1) in 56.7% (34/60) of patients, while conventional imaging (CT + bone scintigraphy) did so only in 16.7% (10/60) (*p* < 0.001). PET/CT’s detection rate was higher than that of conventional imaging in all anatomical regions: pelvic lymph nodes (N) 45% vs. 13.3% (*p* < 0.001), extraregional lymph nodes (M1a) 11.7% vs. 1.7% (*p* = 0.03), and bone metastases (M1b) 23.3% vs. 6.7% (*p* = 0.002). These results are presented in Figure 3. As a consequence, no patients were understaged by ^68^Ga-PSMA-11 PET/CT compared to conventional imaging; on the contrary, molecular imaging led to a TNM upstaging in 45.0% (27/60) of patients: 21.7% (13/60) from N0M0 to N1M0, 18.3% (11/60) from N0M0 to M1, and 5% (3/60) from N1M0 to M1. Patients’ staging according to CI and PSMA-PET/CT is reported in Figure 4. Among the patients with newly detected distant metastases (M1, *n* = 14), exclusive skeletal involvement (M1b) was found in 64.3% (9/14) of cases, followed by exclusive extraregional lymph node involvement (M1a) in 28.6% (4/14) and the involvement of both sites (M1a+b) in 7.1% (1/14) of cases. Regarding disease burden, PSMA-PET/CT detected novel oligometastatic and multimetastatic dissemination in 36.0% (18/50) and 12.0% (6/50) of CI N0M0 patients, respectively; furthermore, novel multimetastatic spread was identified in 25.0% (2/8) of patients with CI oligometastatic disease (N/M1).

With regard to patient management, PET/CT had the potential to change management in 28.8% (17/59) of patients staged with conventional imaging. Specifically, a systemic treatment strategy could have been adopted in 10 patients due to previously unknown PSMA-positive multi-metastatic dissemination; the lymphadenectomy approach could have been modified in three patients due to the identification of pathologic lymph nodes outside the standard surgical template; SABR treatment could have been considered in three patients with oligometastatic disease; in one patient, PSMA-PET/CT identified an unknown collateral oncologic finding unrelated to prostate cancer (urothelial cancer). These data are summarized in Table 2. Moreover, five additional patients exhibited false positive skeletal findings at bone scintigraphy (based on follow-up clinical/imaging data); these patients were correctly staged by PSMA-PET/CT, potentially obviating the need for further confirmatory tests and thereby saving time and costs.

### 3.6. PSMA-PET/CT Accuracy in Pelvic Lymph Node Staging

The accuracy of ^68^Ga-PSMA-11 PET/CT in pelvic lymph node staging was evaluated using the postoperative histopathology data of 32 patients as a reference standard. The analysis was conducted both on a per-patient and per-region (hemipelvis) basis.

At the per-patient analysis, the sensitivity, specificity, positive predictive value, negative predictive value, and accuracy for detection of pelvic nodal metastases were 92.3% [95%IC: 64.0–99.8], 89.5% [66.9–98.7], 85.7% [61.6–95.7], 94.4% [72.0–99.1], and 90.6% [75.0–98.0], respectively. In 6.3% (2/32) of patients, the PSMA-PET/CT nodal findings were not confirmed at histopatology and thereby labeled as false positive results. However, one patient had a nodal finding of uncertain interpretation at the PET/CT scan (PSMA-RADS 3), while another had a focal uptake in a deep pelvic nodal region that was difficult to access laparoscopically and showed post-surgical biochemical persistence. The per-region (hemipelvis) analysis also revealed a comparable accuracy of 89.1% [78.8–95.5]. Data regarding PSMA-PET/CT nodal staging accuracy are reported in detail in Table 3.

### 3.7. Biochemical Response after Radical Prostatectomy

Follow-up data after the radical prostatectomy were available in 29 patients. In the subcohort (*n* = 23) with concordance between conventional and molecular imaging results at staging (i.e., no additional distant or loco-regional metastases outside the surgical template at PSMA-PET/CT), 73.9% (17/23) of patients achieved a complete biochemical response after radical prostatectomy and lymphadenectomy, while 26.1% (6/23) had biochemical persistence. Among those with persistence, five had positive surgical margins, and one showed neuroendocrine differentiation, which can limit PSMA-PET/CT sensitivity.

In the subcohort of patients (*n* = 6) with additional distant or loco-regional metastases identified at staging PSMA-PET/CT, 83.3% (5/6) had biochemical persistence and the follow-up PSMA-PET/CT scan confirmed the nodal/skeletal metastases previously identified at staging. Only one patient showed a complete biochemical response despite a suspicious skeletal metastasis at staging PSMA-PET/CT, which, however, was reported as equivocal (PSMA-RADS 3).

## 4. Discussion

The primary staging of high-risk PCa patients has traditionally relied on the use of abdominal-pelvic CT and bone scintigraphy for the detection of lymph nodal and distant metastases. However, these imaging techniques have intrinsic limitations in terms of sensitivity and specificity. Molecular imaging with PSMA-PET/CT has emerged as an accurate imaging modality for the detection of PCa localizations in different clinical settings, including the primary staging of high-risk PCa patients. However, the diagnostic advantage of PSMA-PET/CT in relation to patients’ clinical characteristics, its accuracy compared to histopathology, and its impact on patient management are still a matter of debate. Therefore, this prospective study, which reflects real-world practice, aimed to evaluate the ^68^Ga-PSMA-11 PET/TC diagnostic performance in the primary staging of patients with high-risk PCa compared to conventional imaging modalities, as well as to assess its potential impact on patient management. Furthermore, the diagnostic accuracy of ^68^Ga-PSMA-11 PET/CT in pelvic nodal staging was assessed using postoperative histopathology data as a reference standard.

In our study, PSMA-PET/CT identified a significantly higher proportion of metastatic patients compared to conventional imaging (CI) (56.7% vs. 16.7%), both in terms of locoregional nodal metastases (N1M0, 25% vs. 8.3%) and distant metastases (M1, 31.7% vs. 8.3%); overall, molecular imaging led to a TNM upstaging in 45.0% (27/60) of patients. The present results about PET/CT detection rates are in line with the study of Zacho et al. [14], reporting a 46% rate of metastatic localizations (N1 and/or M1) and a 27% prevalence of scans positive for locoregional nodal invasion in high-risk PCa patients. As for distant metastatic localizations, the multicenter prospective study by Roach et al. [15] on 108 intermediate and high-risk PCa patients reported a lower detection rate (6%); however, the variability in the detection rate compared to the present study could be due to the different cohort characteristics. Indeed, our real-life study population is characterized by a higher a priori probability of metastatic localizations with respect to the intermediate-risk PCa subgroup included by Roach and colleagues. More recently, a study by Luining et al. [16] on 1879 EAU high-risk PCa patients (mainly scanned with ^18^F-DCFPyL and ^68^Ga-PSMA-11) reported an overall scan positivity rate of 45%, with a 10% prevalence of locoregional disease and a 35% rate of distant metastatic involvement. Interestingly, they also showed that the prevalence of metastatic disease can range widely between 20% and 60% among high and very-high PCa risk-groups, thus highlighting the potential benefit of further stratifying the prognostic groups (as in the NCCN-National Comprehensive Cancer Network or CPG-Cambridge Prognostic Group classifications). At present, this hypothesis could not be tested in our prospective cohort due to the limited sample size, which prevented further stratification.

With regard to the comparative performance of PSMA-PET/CT and CI, the results of our analysis are consistent with other literature studies, where ^68^Ga-PSMA-11 PET/CT was shown to outperform CI. In the prospective multicenter randomized proPSMA trial [7] on 302 high-risk PCa patients, PSMA-PET/CT showed a 27% greater accuracy than conventional imaging (92% vs. 65%), with superior performance in the evaluation of both pelvic nodal and distant metastases. Multiple studies have later confirmed the higher diagnostic accuracy of molecular imaging, as shown in the metanalysis by Chow et al. [17], where PSMA-PET achieved a significantly higher sensitivity (73.2% [95% CI: 56.4–85.2] vs. 38.5% [31.9–45.5]) and specificity (97.8% [96.0–98.8] vs. 83.6% [73.3–90.4]) than CT in the nodal staging of 687 patients. Similarly, the reported sensitivities and specificities of PSMA-PET versus BS for skeletal staging (541 patients) were 98.0% (88.0–99.7) versus 73.0% (63.6–80.7) and 96.2% (90.9–98.5) versus 79.1% (72.3–84.4), respectively. As for pelvic nodal staging, the OSPREY trial [3], which analyzed 252 high-risk PCa patients, showed ^18^F-DCFPyL PET/CT to have a threefold higher PPV (86.7% vs. 28.3%), a significantly higher specificity (97.9% vs. 65.1%), and a slightly higher NPV (83.2% vs. 77.8%) compared to CI. Moreover, PET/CT detected extra-pelvic lesions in 12.3% of patients, upstaging them from clinical M0 to M1. Similarly, in our study, 18.3% (11/60) of patients with negative conventional imaging were upstaged to M1 due to the identification of extra-pelvic lesions. Beyond ^68^Ga-PSMA-11 and ^18^F-DCFPyL, the superior detection rate of nodal disease was also confirmed using other PSMA-based tracers, such as ^18^F-PSMA-1007. Indeed, in the study by Malaspina et al. [18] on 79 intermediate and high-risk PCa patients, pelvic nodal localizations were detected by PSMA-PET/CT and CT in 34.2% and 10.1% of patients, respectively, resulting in a patient-level sensitivity of 87% (95% CI: 71–95) vs. 37% (22–55).

In the present analysis, BS detected suspicious bone metastases in 16.7% (10/60) of patients, with a performance comparable to the 12–14% BS detection rate reported by Shanmugasundaram et al. [19] and Luining et al. [16] in EAU high-risk PCa patients. In accordance with the known limited specificity of BS, skeletal involvement was confirmed only in 4/10 patients. Indeed, BS has been reported to show more equivocal lesions than PSMA-PET/CT (15.9% vs. 1.4%) in previous literature studies [20], leading to higher FP rates (16.0–34.8% vs. 0–11.8%) [17]. PSMA-PET/CT could thus potentially lower the risk of over-staging and/or treatment delay due to further testing. However, it is well known that PSMA-PET/CT performance in skeletal staging can be affected by the specific radiotracer. In fact, higher FP rates are known to occur with ^18^F-PSMA-1007. In our study with ^68^Ga-labelled PMSA, PET/CT was able to identify novel skeletal metastases undetected at BS in 15% (9/60) of patients with no cases of understaging, thus confirming its previously reported higher detection rates [21].

Overall, in our cohort, ^68^Ga-PSMA-11 PET/CT led to a TNM upstaging in 45.0% (27/60) of patients. This finding is aligned with other literature studies, where ^68^Ga-PSMA-11 PET/CT was found to modify intermediate and/or high-risk PCa patients’ staging compared to CI in a range of 28% to 55% [7,14,22,23,24].

Globally, in our study, ^68^Ga-PSMA-11 PET/CT had the potential to change management in 28.8% (17/59) of patients staged with CI. This is in agreement with previous studies where the reported rate of change in management ranged between 21% and 28% [7,15,25], as well as with the results of a later metanalysis [26] on 1099 patients, which reported a 28% (95% CI: 23.0–34.0%) rate.

In our study, the great majority of pathologic findings at PSMA-PET/CT had a PSMA-RADS score ≥4, showing a good reader confidence in the scans evaluation. This is in line with the data reported by the proPSMA trial [7], in which PET/CT was associated with a lower rate of equivocal findings compared to CI (7% vs. 23%) and with a higher inter-reader agreement (κ = 0·87 for nodal and κ = 0·88 for distant metastases). Similarly, a following metanalysis reported a higher inter-reader agreement for PSMA-PET (0.78–0.92) than for CI (0.40–0.55) across four studies [3,18,27,28].

Our study also investigated the diagnostic accuracy of ^68^Ga-PSMA-11 PET/CT in pelvic nodal staging compared to postoperative histopathology data. The per-patient sensitivity, specificity, PPV, and NPV were 92.3% (95%IC: 64.0–99.8), 89.5% (66.9–98.7), 85.7% (61.6–95.7), and 94.4% (72.0–99.1), respectively. Comparable results have been previously reported by the proPSMA trial [7], where ^68^Ga-PSMA-11 PET/CT achieved a sensitivity and specificity of 85% (74–96) and 98% (95–100), significantly higher than those of CI (38% [24–52] and 91% [85–97], respectively). A more recent metanalysis, however, reported lower values in high-risk PCa patients, with sensitivity, specificity, PPV, and NPV values of 54% (95% CI: 37–70), 95% (91–98), 77% (67–86), and 83% (79–87) [29]. The findings of the above metanalysis were later confirmed by other studies using histopathology as a reference standard, such as the prospective multicenter phase-3 trial by Hope et al. [30] on 277 surgically treated patients with intermediate/high-risk PCa. The per-patient sensitivity, specificity, PPV, and NPV for pelvic nodal metastases were 40% (34–46), 95% (92–97), 75% (70–80), and 81% (76–85), respectively. As stated by the authors, the sensitivity reported by the proPSMA trial was not comparable to these results since the former used a composite endpoint with multiple criteria other than histopathology. The higher sensitivity reported in our study could also partly be a result of the limited sample size of our cohort. Indeed, although PSMA PET/CT is more sensitive in nodal staging and recent technological advancements have improved the scanners’ performance [31,32,33], small nodal metastases under the spatial resolution of PET scanners may still be missed. Considering an NPV of approximately 80%, 20% of patients taken to prostatectomy with a negative PET will have nodes on pathology; thus, a negative PET cannot be used as a rule-out criterion for pelvic nodal dissection. However, PET imaging is already used in risk calculators and nomograms to predict nodal disease—as in the Amsterdam–Brisbane–Sydney nomogram [34]—and has shown an intrinsic prognostic value [35]. As for false positive results, as previously reported [30], those findings may also partially be attributed to residual disease not identified after lymphadenectomy. Finally, beyond ^68^Ga-PSMA-11, comparable diagnostic performances in nodal staging were also reported by studies using ^18^F-DCFPyL such as the OSPREY [3] and the SALT trials [36]. Specifically, the OSPREY trial investigated a cohort of 252 high-risk PCa patients reporting a median sensitivity, specificity, PPV, and NPV of 40.3% (28.1–52.5%), 97.9% (94.5–99.4%), 86.7% (69.7–95.3%), and 83.2% (78.2–88.1%), respectively.

When comparing molecular imaging to CI, further aspects should also be taken into account, such as diagnostic radiation exposure and cost-effectiveness. Indeed, aside from its higher diagnostic performance, PSMA-PET/CT could also allow for significantly reducing the radiation exposure compared to CI, as reported by Hofman et al. (8.4 vs. 19.2 mSv) [7,37]. Considering the improved diagnostic accuracy and the reduced diagnostic radiation exposure, PSMA-PET/CT has the potential for increased cost-effectiveness compared to CI: indeed, from a patient and healthcare perspective, the increased costs of molecular imaging could be balanced by the benefits and cost savings resulting from a more accurate disease staging, a more effective image-guided approach, an improved quality of life, and the avoidance of unnecessary treatments [38,39]. A previous cost-effectiveness analysis in the Australian setting has demonstrated an advantage for PSMA-PET/CT over conventional imaging [40], but more robust confirmatory data are still needed. Recently, a preliminary cost-effectiveness analysis in the USA and European settings (Belgium, Germany, Italy, and the Netherlands) has also been published [41].

Finally, in parallel with the future larger adoption of PSMA-PET/CT in the primary staging of prostate cancer, further research should also be aimed towards exploring the impact of different PSMA radiotracers on patients’ outcomes. Indeed, in a recent study by Bauckneht et al. [42], different imaging modalities were shown to significantly influence the outcomes of a cohort of 402 oligorecurrent PCa patients undergoing MDT: specifically, ^68^Ga-PSMA-11 PET/CT-guided MDT demonstrated longer PFS (HR: 0.51, 95% CI: 0.26–1.00) and PFS2 (HR: 0.24, 95% CI: 0.09–0.60) compared to ^18^F-PSMA-1007 PET/CT-guided MDT, as well as longer PFS, PFS2, and OS compared to choline-PET/CT-guided MDT.

### Limitations

This study is not exempt from limitations. First, the study cohort comprised 60 patients, and postoperative histopathology data were available only in a subgroup of 32 patients. However, the prospective design of the study and the histopathological validation of PSMA-PET/CT nodal findings represent strengths. Another limitation is the inability to obtain a histopathological validation of all PET/CT findings due to ethical and practical reasons; this aspect, together with the unavailability of composite follow-up data, limits the evaluation of the diagnostic accuracy. Nevertheless, it should be considered that all images were independently interpreted and reported by two nuclear medicine physicians following established guidelines [11], with good agreement and good confidence, as reflected by the PSMA-RADS values. Finally, although PSMA-PET has shown superior diagnostic capabilities over CI, larger prospective multicenter trials are still needed to further investigate whether the PSMA-PET/CT’s impact on patients’ management translates to improved longitudinal oncological outcomes.

## 5. Conclusions

^68^Ga-PSMA-11 PET/TC showed a high diagnostic performance in the primary staging of high-risk prostate cancer, with a higher detection rate compared with conventional imaging, leading to a significant TNM upstaging and potential management change. At pelvic nodal staging, the PSMA-PET/CT findings showed a good correlation with the histopathology data.

## Figures and Tables

**Table 1:** Characteristics of the study population (*n* = 60).

Clinical Parameters		Median	IQR
Age (years)		73	68–76
Prebiopsy PSA (ng/mL)		10.10	6.22–17.95
PSA at PET/CT (ng/mL)		10.51	6.50–21.00
MRI–max diameter (mm)		20.0	15.0–29.0
		**Frequency n (%)**	
MRI–stage ≥ cT3a		22/47 (46.8%)	
MRI–PI-RADS	5	39/47 (83%)	
	4	7/47 (14.9%)	
	3	1/47 (2.1%)	
ISUP ≥ 4		48/60 (80%)	

IQR: interquartile range; ISUP: International Society of Urological Pathology; MRI: magnetic resonance imaging; PET/CT: positron-emission tomography/computed tomography; PI-RADS: Prostate Imaging–Reporting and Data System; PSA: prostate-specific antigen.

**Table 2:** Potential change in management driven by PSMA-PET/CT.

Change in Management	Frequency % (n)
Switch to systemic therapy for newly discovered multimetastatic spread	16.9% (10/59)
Change in lymphadenectomy template in patients who are candidates for surgery	5.1% (3/59)
Potential SABR treatment for oligometastatic disease	5.1% (3/59)
Identification of collateral PSMA-avid oncologic findings	1.7% (1/59)

PSMA: prostate-specific membrane antigen; SABR: stereotactic ablative radiotherapy.

**Table 3:** Validation of ^68^Ga-PSMA-11 PET/CT pelvic nodal staging accuracy compared with post-operative histopathology data.

	Per-Patient Analysis % [95% CI]	Per-Region Analysis % [95% CI]
Sensitivity	92.3% [64.0–99.8]	85.7% [57.1–98.2]
Specificity	89.5% [66.9–98.7]	90.0% [78.1–96.7]
Positive predictive value	85.7% [61.6–95.7]	70.6% [50.4–85.0]
Negative predictive value	94.4% [72.0–99.1]	95.7% [86.1–98.8]
Accuracy	90.6% [75.0–98.0]	89.1% [78.8–95.5]

## Data Availability

The datasets presented in this article are not readily available because the data are part of an ongoing study.
